# Supplementary material for: LbCas12a-D156R Efficiently Edits LOB1 Effector Binding Elements to Generate Canker-Resistant Citrus Plants
Source: Cells. 2022 Jan 18;11(3):315. doi: 10.3390/cells11030315 (PMC8834406; doi:10.3390/cells11030315)
Supplement: Supplementary file 1 [file cells-11-00315-s001.zip › cells-1541542-supplementary.pdf]

GGATCCATGAGCAAGCTCGAGAAGTTTACCAACTGCTACAGCCTCTCTAAGACCCTCAGGTTCAAGGCTATCCCTGTGGGAAAGACCCAAGAGAATATCGACAA
CAAGAGGCTCCTCGTCGAGGATGAGAAGAGAGCTGAAGATTACAAGGGCGTGAAGAAGCTCCTCGACAGGTACTACCTCAGCTTCATCAACGATGTGCTCCACA
GCATCAAGCTCAAGAACCTCAACAACTACATCAGCCTCTTCCGTAAGAAAACCAGGACCGAGAAAGAGAACAAAGAGCTTGAGAACCTCGAGATCAACCTCCGT
AAAGAGATCGCCAAGGCTTTCAAGGGAAACGAGGGATACAAGAGCCTCTTCAAGAAGGATATTATCGAGACAATCCTGCCTGAGTTCCTGGACGATAAGGATGA
GATCGCTCTCGTGAACAGCTTCAACGGATTCACTACTGCCTTCACCGGATTCTTC**CGT**AACAGGGAAAACATGTTCAGCGAAGAGGCCAAGAGCACCTCTATCGC
TTTCAGATGCATCAACGAGAACCTCACGCGTTACATCAGCAACATGGACATCTTCGAGAAGGTGGACGCCATCTTCGATAAGCACGAGGTGCAAGAAATCAAAG
AGAAGATCCTCAACAGCGACTACGACGTCGAGGACTTTTTTGAAGGGGAGTTCTTCAACTTCGTTCTCACCCAAGAGGGCATCGACGTGTACAACGCTATTATCG
GAGGATTCGTGACCGAGTCTGGGGAGAAGATTAAGGGACTCAACGAGTACATCAACCTGTACAACCAGAAAACGAAGCAGAAGCTCCCGAAGTTCAAGCCGCT
CTACAAGCAGGTTCTCTCTGATCGTGAGAGCCTCTCATTTTACGGTGAGGGTTACACCTCTGACGAGGAAGTGCTTGAGGTTTTCCGTAACACCCTCAACAAGAA
CAGCGAGATCTTCTCGTCCATCAAGAAGTTGGAGAAGCTTTTCAAGAACTTCGACGAGTACAGCAGCGCTGGGATCTTCGTTAAGAACGGACCTGCTATCAGCAC
CATCAGCAAGGATATTTTCGGCGAGTGGAACGTGATCAGGGACAAGTGGAATGCTGAGTACGATGACATCCACCTCAAGAAGAAGGCTGTCGTCACTGAGAAGT
ACGAGGATGACAGGCGTAAGTCGTTCAAGAAGATCGGCTCTTTCAGCCTCGAGCAGCTTCAAGAATACGCTGATGCTGATCTCAGCGTGGTCGAGAAGCTCAAA
GAGATCATCATCCAGAAGGTCGACGAGATCTACAAGGTGTACGGGTCCTCTGAGAAGTTGTTCGATGCTGATTTCGTCCTCGAGAAGAGTCTGAAGAAGAACGA
CGCTGTCGTCGCGATCATGAAGGATTTGCTCGACAGCGTGAAGTCCTTCGAGAACTATATCAAGGCCTTCTTCGGAGAGGGCAAAGAGACTAATAGGGACGAGT
CTTTCTACGGGGATTTCGTGCTCGCTTACGATATCCTCCTCAAGGTGGACCATATCTACGACGCCATCAGAAACTACGTGACCCAGAAGCCTTACAGCAAGGACA
AGTTCAAGTTGTACTTTCAGAACCCGCAGTTCATGGGCGGATGGGACAAAGACAAAGAGACAGATTACAGGGCCACCATCCTCAGGTACGGGTCTAAGTACTAC
CTGGCCATCATGGACAAGAAATACGCCAAGTGCCTCCAAAAGATCGACAAGGATGACGTGAACGGGAACTATGAGAAGATCAACTACAAGCTCCTTCCGGGACC
GAACAAGATGCTTCCTAAGGTGTTCTTCAGCAAGAAATGGATGGCCTACTACAACCCGTCTGAGGACATCCAGAAAATCTACAAGAACGGGACCTTCAAGAAAG
GCGACATGTTCAACCTCAACGACTGCCACAAGCTCATCGATTTCTTCAAGGACAGCATCTCGCGTTACCCGAAGTGGTCTAACGCTTACGACTTTAACTTCAGCG
AGACAGAAAAGTACAAGGATATCGCCGGGTTCTACCGTGAGGTTGAGGAACAGGGTTACAAGGTTAGCTTCGAGAGCGCCTCCAAGAAAGAGGTTGACAAGTT
GGTCGAAGAGGGCAAGCTCTACATGTTCCAGATCTATAACAAGGACTTCTCCGACAAGAGCCACGGAACTCCTAACCTCCATACGATGTACTTCAAGCTGCTTTT
CGACGAGAACAACCACGGGCAGATCAGACTTTCTGGTGGTGCTGAACTCTTCATGCGTAGGGCCTCACTCAAGAAAGAAGAGTTGGTTGTTCACCCGGCCAACTC
TCCAATCGCTAACAAGAATCCTGACAACCCGAAAAAGACCACCACGCTGTCTTACGACGTCTACAAGGACAAAAGGTTCAGCGAGGACCAGTACGAGCTTCATA
TCCCGATCGCTATCAACAAGTGCCCGAAGAACATCTTCAAGATCAATACCGAGGTGAGGGTGCTGCTCAAGCACGATGATAACCCTTACGTGATCGGAATCGATC
GTGGTGAGAGAAACCTCCTCTACATCGTTGTGGTGGACGGAAAGGGAAACATCGTCGAGCAGTACAGCCTGAACGAGATTATCAACAATTTCAACGGCATCAGG
ATCAAGACCGACTACCACTCACTCCTCGATAAGAAAGAAAAAGAGCGTTTCGAGGCCAGGCAGAACTGGACTTCTATCGAAAACATCAAAGAGTTGAAGGCCGG
CTACATCTCTCAGGTGGTGCATAAGATCTGCGAGCTGGTGGAAAAGTACGATGCTGTGATCGCTCTTGAGGACCTCAACTCTGGGTTCAAGAACAGTAGAGTGAA
GGTTGAGAAGCAGGTCTACCAAAAGTTCGAGAAGATGCTCATCGACAAGCTCAACTACATGGTGGACAAAAAGAGCAACCCTTGCGCTACCGGTGGTGCTCTTA
AGGGATACCAGATCACGAACAAGTTCGAGTCCTTCAAGAGCATGAGCACCCAGAACGGCTTCATCTTCTATATCCCTGCTTGGCTCACCAGCAAGATCGATCCTT
CTACTGGTTTCGTGAACCTGCTCAAGACCAAGTACACCTCGATCGCCGACAGCAAGAAGTTCATCTCGTCTTTCGACAGGATCATGTACGTGCCGGAAGAGGATC
TTTTCGAGTTCGCTCTCGACTATAAGAACTTCAGCAGGACCGACGCCGACTACATTAAGAAGTGGAAGCTCTACTCCTACGGGAACCGTATCAGGATCTTCCGAA
ATCCGAAGAAAAACAACGTGTTCGACTGGGAAGAAGTGTGCCTCACCTCTGCCTACAAAGAACTGTTCAACAAGTACGGCATCAACTACCAGCAGGGTGATATC
AGGGCTCTTTTGTGCGAGCAGAGCGACAAGGCATTCTACAGCTCATTCATGGCCCTCATGTCTCTCATGCTCCAGATGAGGAACTCTATCACCGGAAGGACCGAT
GTGGACTTCCTTATCTCTCCGGTCAAGAACTCTGACGGGATCTTCTACGACAGCCGTAACTATGAGGCTCAAGAGAACGCTATCCTGCCGAAGAATGCTGATGCA
AACGGGGCTTACAACATTGCGAGAAAGGTTCTCTGGGCTATCGGGCAGTTTAAGAAAGCGGAAGATGAGAAGCTGGACAAGGTGAAGATCGCCATCTCCAACA
AAGAGTGGCTTGAGTACGCTCAGACCTCCGTTAAGCACAAGAGGCCTGCTGCTACTAAGAAAGCTGGTCAGGCTAAGAAGAAGAAATAAGAATTC

Supplementary Figure S1. ttLbCas12a sequence. A temperature-tolerant LbCas12a variant (ttLbCas12a) harbors the single mutation D156R, which is indicated in purple. Restriction Digestion Enzyme sites *Bam*HI and *Eco*RI are underlined. Translation start codon and end codon are highlighted in green and red, respectively.

(A) <u>CTCGAG</u>TCGTTGAACAACGGAAACTCGACTTGCCTTCCGCACAATACATCATTTCTTCTTAGCTTTTTTTCTT
CTTCTTCGTTCATACAGTTTTTTTTTGTTTATCAGCTTACATTTTCTTGAACCGTAGCTTTCGTTTTCTTCTTTT
TAACTTTCCATTCGGAGTTTTTGTATCTTGTTTCATAGTTTGTCCCAGGATTAGAATGATTAGGCATCGAACC
TTCAAGAATTTGATTGAATAAAACATCTTCATTCTTAAGATATGAAGATAATCTTCAAAAGGCCCCTGGGAAT
CTGAAAGAAGAGAAGCAGGCCCATTTATATGGGAAAGAACAATAGTATTTCTTATATAGGCCCATTTAAGTT
GAAAACAATCTTCAAAAGTCCCACATCGCTTAGATAAGAAAACGAAGCTGAGTTTATATACAGCTAGAGTC
GAAGTAGTGATTGAAATTACTGATGAGTCCGTGAGGACGAAACGAGTAAGCTCGTCTAATTTCTACTAAGT
GTAGATCTTTCTCTATATAAACCCCTTTTGGCCGGCATGGTCCCAGCCTCCTCGCTGGCGCCGGCTGGGCAA
CATGCTTCGGCATGGCGAATGGGACTTTTT<u>GAGCTC</u>

(B) ACACCTTGGTAATTTTGACATTAGGTAGCAATATAATACGATAAAATTCACCTCCATGTAATTTGAAGTTCTTT
TCAATAATTTTTTTGACAAATTTTATAGAAGAATTTAACCTTTTCTTTTTTTTTTTCAAACGAAGAAATGTTTT
CGTCATTCAATTAAAATTAATGACATCATCTAGTGGCTTGGTGACATACGCTTTAGATACAATTGTCATTCTTG
CCTTTTCCTTTCTCTATATAAACCCCTTTTGCCTTAACTTTGTTTCAACTAAAGCAGCTCCTCCTCATCCCTTA
CTGTCTTCGCTTTCTCACTAACTACTACAACCCAACAGTTTTCTTCTCTCAAAA

Supplementary Figure S2. Part of pUC57-AtU6-26-crRNA:LOBP sequence (A) and Type II CsLOBP in Pummelo (B). Hammerhead ribozyme and hepatitis delta virus ribozyme are shown in yellow and gray, respectively. EBE_{PthA4}-CsLOBP is indicated in red. Artificial dTALE dCsLOB1.5 binding site is highlighted in green. The crRNA targeting site is indicated by blue rectangle. Enzyme sites *Xho*I and *Sac*I are underlined.

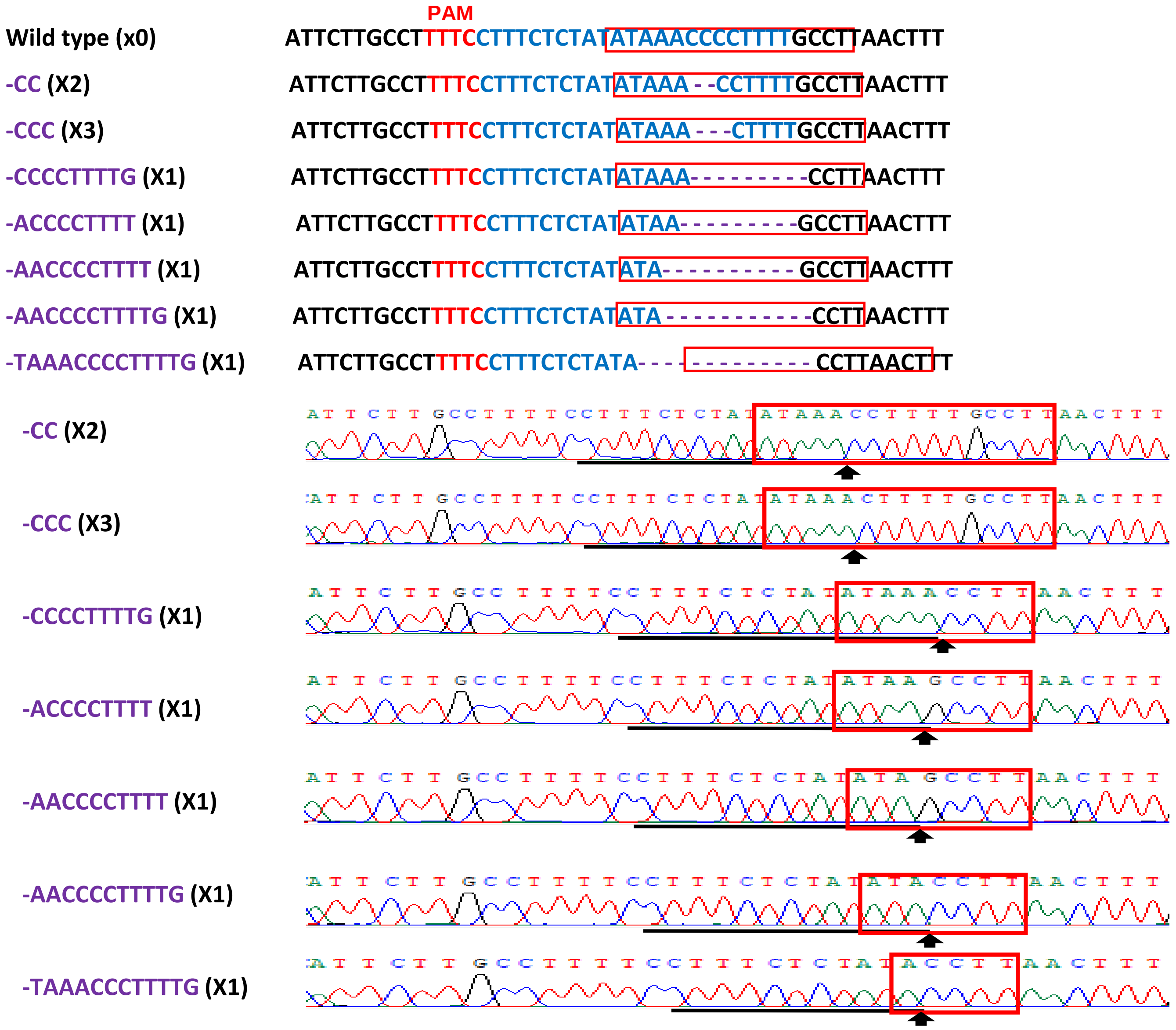

Supplementary Figure S3. Sanger sequencing results of LOBP-edited Pummelo line #Pum$_{tt}$1. Using #Pum$_{tt}$1 genomic DNA as template, PCR products were amplified with LOBP2 and LOBP5. After ligation, 10 colonies were randomly selected, sequenced and analyzed. The targeted sequence is shown in blue, and the mutations are shown in purple. EBE$_{PthA4}$-CsLOBP is highlighted by red rectangles. The targeted sequence is underlined by black lines, and the mutant sites are indicated with arrows.

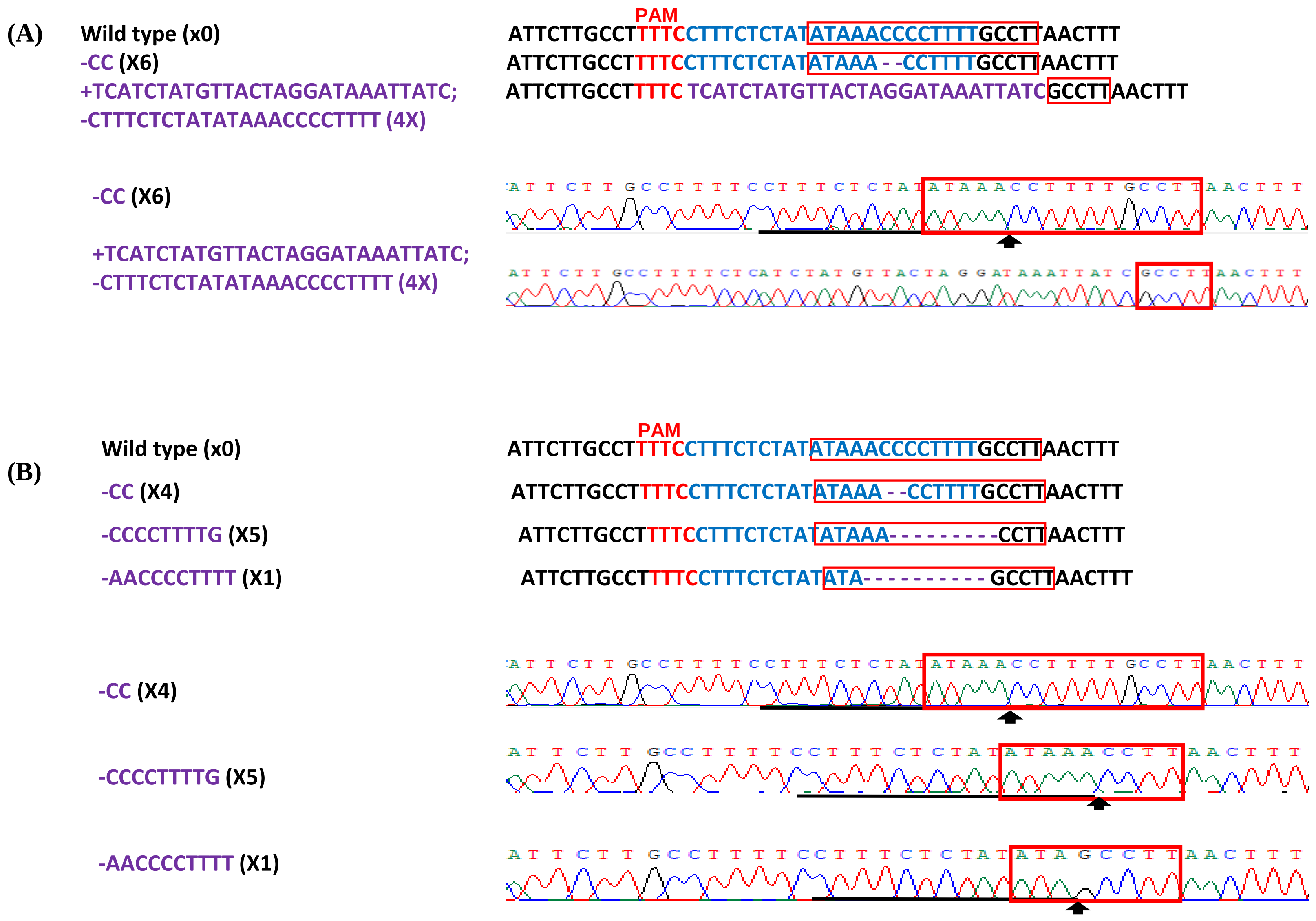

Supplementary Figure S4. Sequence results of LOBP-edited Pummelo lines #Pum$_{tt}$3 (A) and #Pum$_{tt}$4 (B). Using genomic DNA of #Pum$_{tt}$3 or #Pum$_{tt}$4 as template, PCR products were amplified with LOBP2 and LOBP5. After ligation, 10 colonies were randomly selected, sequenced and analyzed. The targeted sequence is shown in blue, and the mutations are shown in purple. EBE$_{PthA4}$-CsLOBP is highlighted by red rectangles. The targeted sequence is underlined by black lines, and the mutant sites are indicated with arrows.

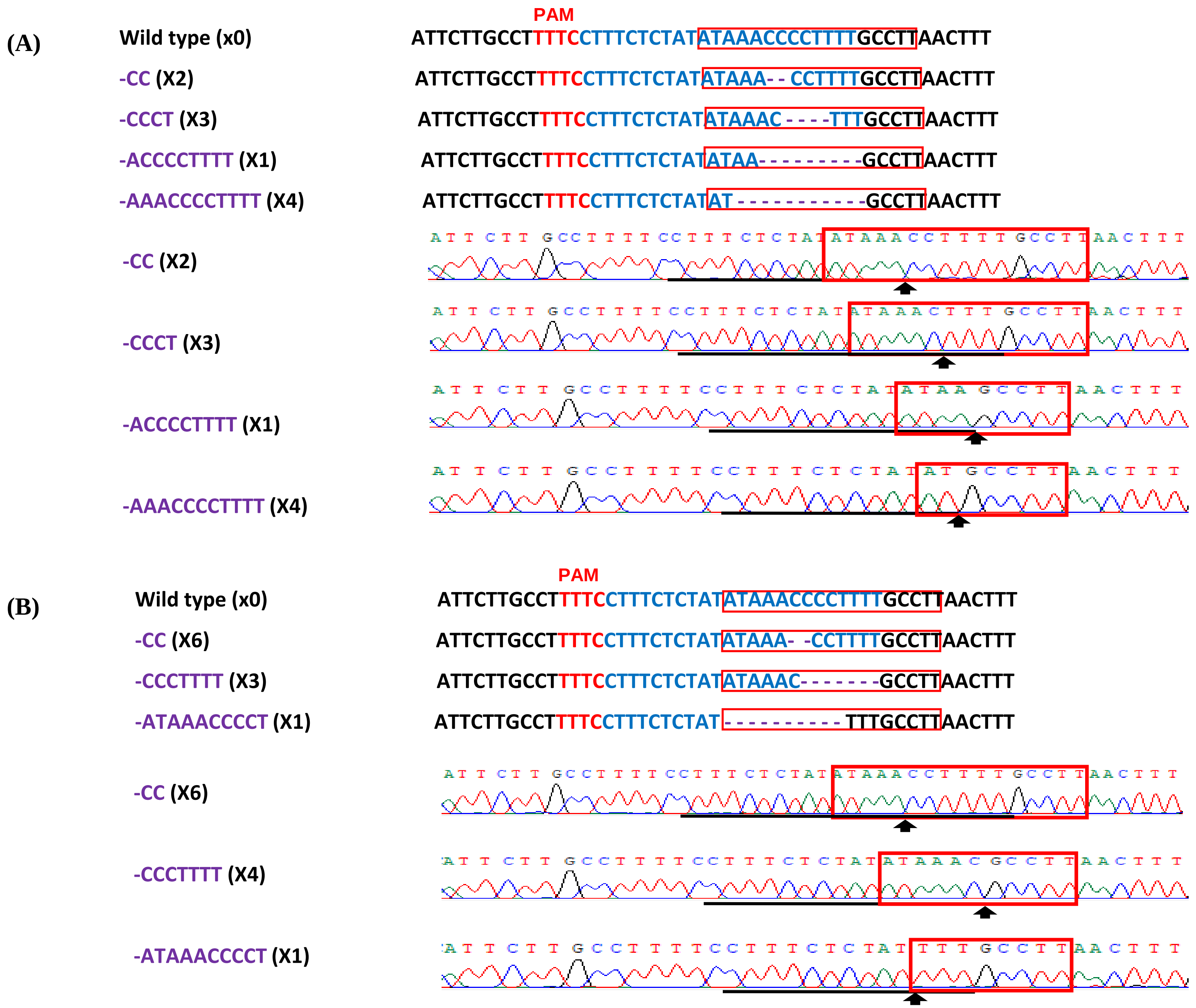

Supplementary Figure S5. Sequence results of LOBP-edited Pummelo lines #Pum$_{tt}$5 (A) and #Pum$_{tt}$6 (B). Using genomic DNA of #Pum$_{tt}$5 or #Pum$_{tt}$6 as template, PCR products were amplified with LOBP2 and LOBP5. After ligation, 10 colonies were randomly selected, sequenced and analyzed. The targeted sequence is shown in blue, and the mutations are shown in purple. EBE$_{PthA4}$-CsLOBP is highlighted by red rectangles. The targeted sequence is underlined by black lines, and the mutant sites are indicated with arrows.

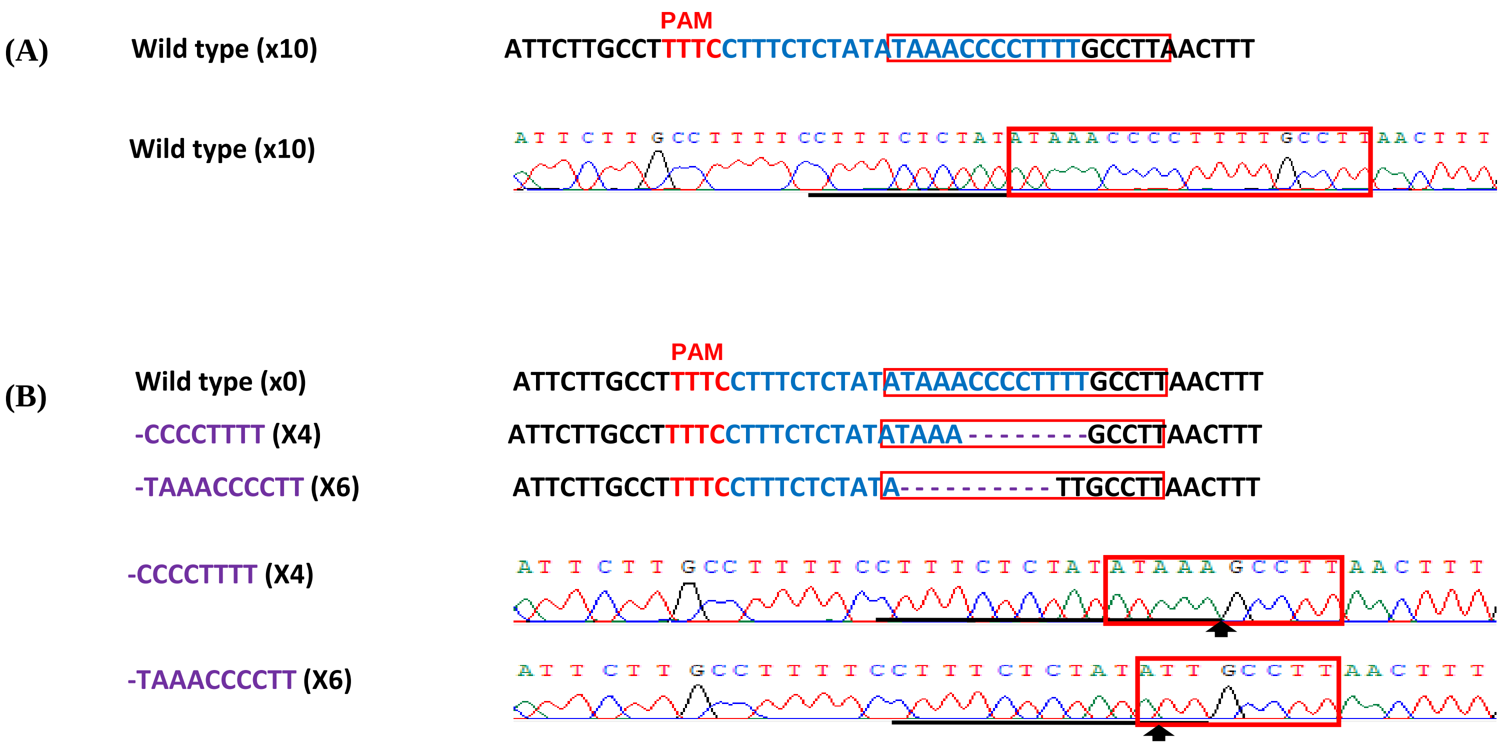

Supplementary Figure S6. Sequence results of LOBP-edited Pummelo lines #Pum$_{tt}$7 (A) and #Pum$_{tt}$8 (B). Using genomic DNA of #Pum$_{tt}$7 or #Pum$_{tt}$8 as template, PCR products were amplified with LOBP2 and LOBP5. After ligation, 10 colonies were randomly selected, sequenced and analyzed. The targeted sequence is shown in blue, and the mutations are shown in purple. EBE$_{PthA4}$-CsLOBP is highlighted by red rectangles. The targeted sequence is underlined by black lines, and the mutant sites are indicated with arrows.

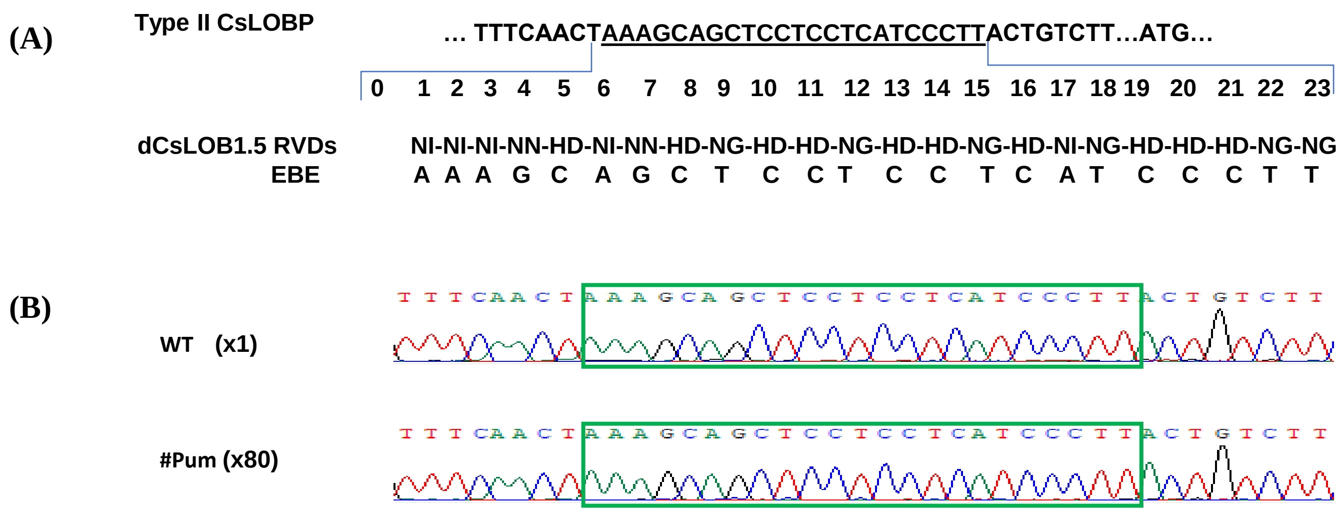

Supplementary Figure S7. dCsLOB1.5 and its representative chromatograms in Pummelo. (A) dCsLOB1.5 is an artificial dTALE, which specifically recognizes AAAGCAGCTCCTCCTCATCCCTT. (B) Representative chromatograms of dCsLOB1.5-binding sequence, which is the same in wild type and transgenic Pummelo plants. The dCsLOB1.5-binding sequence is highlighted by green rectangles.

Bulge Type | DNA bulge | RNA bulge | 3 | Filter | Download filtered result

Bulge Type	Target	Chromosome	Position	Direction	Mismatches	Bulge Size
X	crRNA: TTTVCTTTCTCTATATAAACCCCTTTT<br>DNA: TTTCCTTTCTCTATATAAACCCCTTTT	NC_023052.1	28359397	-	0	0

Supplementary Figure S8. Off-targets of GFP-p1380N-ttLbCas12a:LOBP crRNA. A web software (http://www.rgenome.net/cas-offinder/) was used to analyze off-targets induced by GFP-p1380N-SpCas9p:PumLOBP crRNA. No potential off-targets were identified when up to 3 bp mismatches with the targeting crRNA were used for searching.
